# Minimally invasive pancreatic cancer surgery: What is the current evidence?

**DOI:** 10.1007/s12032-017-0984-4

**Published:** 2017-06-01

**Authors:** Michał Pędziwiatr, Piotr Małczak, Piotr Major, Jan Witowski, Beata Kuśnierz-Cabala, Piotr Ceranowicz, Andrzej Budzyński

**Affiliations:** 1 2nd Department of General Surgery, Jagiellonian University Medical College, Kopernika 21, 31-501 Kraków, Poland; 2 Centre for Research, Training and Innovation in Surgery (CERTAIN Surgery), Kraków, Poland; 3 Department of Diagnostics, Chair of Clinical Biochemistry, Jagiellonian University Medical College, Kraków, Poland; 4 Department of Physiology, Jagiellonian University Medical College, Kraków, Poland

**Keywords:** Pancreatic cancer, Laparoscopy, Minimally invasive surgery, Pancreatoduodenectomy, Whipple, Distal pancreatectomy

## Abstract

Surgery remains the only option to cure pancreatic cancer. Although the use of laparoscopy in oncology is rapidly growing worldwide, its efficacy in pancreatic surgery remains controversial. A number of studies have compared outcomes of minimally invasive and open pancreatic resections. However, they are mostly non-randomized trials including relatively small groups of patients. In addition, most of these studies were conducted in high-volume pancreatic centres. It seems that despite longer operative time, laparoscopy may be beneficial in terms of morbidity, blood loss and hospital stay. Thus far, very little is known about the long-term outcomes of laparoscopic surgery for pancreatic cancer. Our aim was to review current evidence for the use of minimally invasive techniques in patients with pancreatic malignancy.

## Introduction

Pancreatic cancer is one of the most aggressive malignancies associated with extremely poor 5-year survival rate (~6–7%) [1]. Since the tumour remains asymptomatic in the early stage, only 15% of patients have resectable disease at diagnosis [2]. In the remaining cases, local infiltration, invasion of surrounding vessels and the presence of early distant metastases are considered the major causes precluding radical surgical treatment. It has been calculated that in 2016 the rate of pancreatic cancer in European population was around 8/100,000 in men and 5.7/100,000 in women [3]. In a report published in 2016 by Ferlay et al., authors suggest there will be more deaths annually from pancreatic cancer in the European Union countries than deaths from breast cancer. With predicted 91,500 deaths from pancreatic cancer, the disease may become the third most important cause of cancer death in the EU, after lung and colorectal cancers, within the span of a few years [4]. Despite aggressive treatment combining surgery with systemic chemotherapy, the median survival in patients undergoing radical resection estimated for 25–28 months is still unsatisfactory [5]. It also drops below 12 months in unresectable patients treated with palliative chemotherapy/radiotherapy only [1].

Surgery remains the only option to cure pancreatic malignancies. Since first reports by Walther Kausch in 1912 and Allen Whipple in 1935, the operative technique and perioperative care have been gradually improved. Post-operative mortality has decreased from 30% in the early years to 3–5%, as shown in the most recent analyses [6–8]. Nevertheless, pancreatic cancer surgery is still associated with relatively high morbidity of approximately 40% [9].

## Minimally invasive pancreatoduodenectomy (MIPD)

Historically, the first laparoscopic pancreatoduodenectomy (LPD) was performed in 1994 by Michel Gagner, and since then, one of the most complex and demanding abdominal procedures entered the world of minimally invasive surgery [10]. Interestingly, the same author published three years later a series of 10 cases in which the mean operative time was 8.5 h and the conversion rate was 40% [11]. He concluded that although it was feasible, the advantages of a complete laparoscopic Whipple were questionable. There are, however, several well-known benefits of laparoscopic approach, which include: faster recovery, reduced inflammatory response, reduced intraoperative blood loss, less post-operative pain, decreased morbidity and better cosmetic effect. For these reasons, the use of laparoscopy is rapidly growing worldwide. It has been documented that laparoscopic surgery is feasible and safe in other disciplines of oncologic surgery. More than two decades later, minimally invasive pancreatoduodenectomy is still a subject of debate. One can argue whether a 5- to 8-h-long surgical procedure, involving removal of a part of the pancreas, distal stomach, entire duodenum and the gallbladder, can really be called a ‘minimally invasive’ procedure? On the one hand, MIPD does not require long incision, and for this reason, it can indeed be called ‘less invasive’. On the other hand, surgical trauma related to the extent of dissection, regardless of the approach, significant alterations in gastrointestinal anatomy, relatively high morbidity and long hospital stay definitely speak against the minimally invasiveness of this procedure.

A number of systematic reviews compared outcomes of minimally invasive (laparoscopic, hand-assisted or robotic) and open pancreatoduodenectomy. However, many of them include studies comprising pure MIPD and hybrid procedures, where the dissection is performed minimally invasively, but the anastomoses are created manually via minilaparotomy [12–14]. The most recently published meta-analysis included 12 non-randomized studies (only 2 of them were prospective) with a total of 2186 patients (705 underwent MIPD and 1481 underwent open procedure) [15]. Only studies involving pure MIPD (meaning both resection and anastomoses were performed in a minimally invasive fashion) were included. In eight studies, patients underwent robotic pancreatoduodenectomy (RPD), while four papers analysed laparoscopic cases. The operative time was significantly longer in MIPD group (464 vs. 388 min), whereas blood loss and length of stay decreased. Although there were differences in the rate of delayed gastric emptying in the laparoscopic subgroup, it was not different in robotic cases. Pancreatic fistula rate, as well as the overall morbidity, did not vary between groups. In addition, authors analysed oncologic outcomes in patients with pancreatic ductal adenocarcinoma. Resection margin status was extracted from six studies, and a subsequent meta-analysis revealed no significant differences between the groups. R1 resection was present in 62/223 (27.80%) in MIPD cases versus 200/727 (27.51%) in open approach group. Similarly, the number of harvested lymph nodes was not different in laparoscopic nor robotic group in comparison with open approach. The review addressed potential risks of bias, one of them being different size of removed tumours (smaller in MIPD group). In addition, all studies contained patients from high-volume centres, which presented slightly different operative technique in each of them—this might have influenced the final outcomes. The authors concluded that minimally invasive pancreatic head cancer surgery is not ready for general application and should be performed in specialized high-volume pancreatic centres with extensive expertise in minimally invasive surgery.

The data on long-term survival in MIPD are very sparse. Five-year survival in pancreatic cancer, reported by Palanivelu et al., was 32%. However, the majority of included patients had early-stage cancers [16]. Because there are only two comparative studies that show no differences in survival in MIPD versus open surgery for pancreatic head cancer, the results of remaining studies are awaited [17, 18]. Some authors suggested that to improve resectability, the so-called artery first approach (where superior mesenteric artery is dissected in the early phase of resection, before any irreversible step is taken) may be used [19, 20]. In theory, it allows early determination of resectability and decreases R1 resection rate. There are in fact six different approaches to early isolation of the artery, and the choice between them depends on the tumour location and the extent of infiltration [21]. This approach has been shown feasible in laparoscopic surgery as well, however, only in case studies; therefore, firm conclusions about its benefits cannot be drawn [22, 23].

It has been shown that involvement of major veins (superior mesenteric or portal vein) is no longer a contraindication for surgical resection in locally advanced pancreatic cancer. Patients undergoing *en bloc* resection with the involved vein may have similar long-term oncologic survival as compared to those without vascular involvement [24–26]. In borderline tumours, addition of neoadjuvant treatment may be beneficial in both resectability and survival [27]. There are very few pancreatic centres that report minimally invasive vascular resections in pancreatic head surgery [28–30]. Laparoscopic approach is feasible in the hands of an extremely experienced surgeon and may be associated with reduced blood loss and shortened length of stay. Perhaps wider implementation of robotic surgery (wider range of motion, greater accuracy and better ergonomics) will facilitate vascular reconstruction during MIPD. However, it seems that so far open approach will remain the mainstay when it comes to vascular involvement.

It is clear that the outcomes of laparoscopic surgery are related to skill and experience of the surgeon carrying out the procedure as well as the institution and its annual volume.

Previous studies have estimated that the learning curve is at least 50 cases for laparoscopic approach and 33 (up to 80 in one report) for robotic access [31, 32]. MIPD is a demanding procedure, involving meticulous dissection around important vessels, difficult anatomy with a relatively high rate of anatomical abnormalities, long operating time and difficult anastomoses. Currently, there is no standardized surgical training for those who are willing to make their first attempts with MIPD; therefore, full expertise in laparoscopic surgery and open pancreatoduodenectomy is needed. In addition, when analysing outcomes of MIPD, the information on the learning curve is usually not provided, which may bias the results. It has been established that hospital volume correlates with perioperative mortality and it is particularly noticeable in pancreatic surgery [33, 34]. In case of MIPD, Adam et al. [35] estimated that the annual institutional volume of 22 procedures is enough to obtain acceptable outcomes. This, in fact, means that the institutional volume has to be higher, taking into consideration that not every tumour is suitable for MIPD, especially at the beginning of the learning curve.

## Laparoscopic distal (left) pancreatectomy (LDP)

Operations of the body and the tail of the pancreas and pancreatic head surgery are different for many reasons. Firstly, the majority of LDP pancreatectomies are performed for benign tumours, and in these cases, the procedure itself is less extensive, especially in spleen-preserving cases. Because there are no gastrointestinal anastomoses—including pancreatic anastomosis—it literally eliminates one of the major causes of morbidity. On the other hand, cancers of the body and tail of the pancreas are usually diagnosed in a more advanced stage due to asymptomatic course. Malignant cases require appropriate lymphadenectomy (in Gerota’s fascia plane) and splenectomy. This can also be a potential cause of morbidity.

First reports of laparoscopic distal pancreatectomy were made by Sir Alfred Cushieri in 1994 for chronic pancreatitis [36]. Unlike pancreatic head resection, distal pancreatectomy has been adopted more widely. It is now a generally accepted procedure for all benign lesions [37]. According to the recent survey, ¾ of hepatobiliary surgeons have had experience in laparoscopic distal pancreatectomy. Having said that, the median proportion of laparoscopic and open approach per surgeon was still only 30% [38]. Although the enthusiasm for LDP seems to be higher than for the MIPD and is steadily increasing, the evidence for the use of minimally invasive approach in cancer is still very limited. Therefore, surgeons are rather reluctant to adopt LPD in cancer cases. Malignant diagnosis is a contraindication to laparoscopic surgery by every third surgeon, and two-thirds of them would not use LPD in multi-visceral involvement [38].

There have been several meta-analyses so far; however, due to the retrospective nature of included studies and high proportion of benign lesions, all of them are prone to selection bias; therefore, firm conclusions on oncologic benefits cannot be drawn [39]. Looking into results, several potential benefits of minimally invasive access were observed: reduced blood loss, lower morbidity in most of previous meta-analyses (OR 0.71–0.92) and shortened length of hospital stay (by 2.7 up to 12.3 days). Based on the available literature, LPD can be certainly offered to patients with benign lesions and, in the hands of experienced surgeon, may prove to be beneficial. But what about pancreatic cancer cases? In April 2016, a Cochrane review was published by Riviere et al. [40]. It finally included 11 studies (1506 participants: 353 undergoing laparoscopic distal pancreatectomy and 1153 undergoing open distal pancreatectomy for cancer). All of them were cohort or case–control studies with a high risk of bias. The authors concluded that oncologic results were very imprecise and therefore cannot be translated into firm conclusions about feasibility and safety of minimally invasive distal pancreatectomy for cancer. Looking at the most recent systematic review on LDP in pancreatic cancer, only one study reported higher lymph node yield in minimally invasive group, whereas in the remaining nine studies, there were no differences [41]. Patients undergoing laparoscopic procedure were more likely to receive adjuvant chemotherapy earlier (70 vs. 96 days). However, this has not transferred into differences in survival (26 vs. 25 months). It is somehow surprising that more than 20 years after introduction of laparoscopic distal pancreatectomy, with relatively high adoption of this procedure, no randomized controlled trial has been conducted until today. It is even more surprising that in every review on laparoscopic pancreatic resection, the need for a randomized controlled trial is clearly stated and, so far, we encountered only two ongoing projects—the Dutch LEOPARD-1 multicentre randomized controlled trial (http://www.trialregister.nl/trialreg/admin/rctview.asp?TC=5188, estimated completion date 10 April 2017) and the Swedish LAPOP single-centre randomized controlled trial (http://www.isrctn.com/ ISRCTN26912858 estimated completion date in 2020).

In all but one previous meta-analyses (by Nigri et al.), the rate of post-operative pancreatic fistula was comparable regardless of the used technique [39, 42]. Even though a Cochrane review by Probst et al. showed no differences between stapler and scalpel resection, followed by hand-sewn closure of the pancreatic remnant for distal pancreatectomy, for obvious reasons practically all LPDs are performed with the use of linear stapler and the pancreatic fistula rate remains high (30–40%) [43].

Similarly to open surgery, in line with EAES guidelines, spleen-preserving LPD can be considered in patients with benign tumours [37]. Although spleen preservation is associated with longer operative time, it may lead to reduced morbidity (including pancreatic fistula) and shorter LOS [44]. However, the data are contradictory [45, 46]. All these studies comprise relatively small non-randomized cohorts of patients with smaller, benign tumours.

Finally, the introduction of laparoscopy into surgery of the body and tail of the pancreas may have positive impact on post-operative quality of life [47, 48]. At the same time, the direct costs of the surgical procedure are higher comparing to open distal pancreatectomy; however, they can be probably balanced with reduced post-operative costs [37].

## Conclusions

In conclusion, based on the review of available literature, minimally invasive pancreatic surgery for cancer is feasible. In the hands of an experienced surgeon, MIPD can be considered a safe alternative to open approach for benign tumours. However, the evidence on long-term outcomes of minimally invasive access in pancreatic head ductal carcinoma is still sparse. The situation is similar in the case of LPD—it may very well serve as an option to treat tumours located in the body and tail of the organ, but more trials are required to show its oncologic benefits in long-term period. Although the introduction of minimal access may be helpful in terms of length of stay and blood loss, MIPD is still an extensive surgical trauma; thus, other ways to diminish it are still desired. For instance, the introduction of modern perioperative care programmes based on ERAS (enhanced recovery after surgery) principles has been shown to accelerate patients’ recovery, which enables earlier discharge, including open cases [49, 50]. In addition, as demonstrated in other surgical disciplines, thanks to high compliance with ERAS protocol, it is possible to eliminate traditional risk factors for complications and prolonged length of stay [51, 52]. Perhaps, it is also the pathway upon which novel pancreatic surgery is set to venture, along with minimizing the length of incision, with the sole goal of further improving outcomes.
